# Evaluation of a model for providing cost effective, accessible continuing education to the forensic science community

**DOI:** 10.1080/20961790.2021.1928822

**Published:** 2021-08-19

**Authors:** Thomas J. Gluodenis

**Affiliations:** Department of Chemistry & Physics, Lincoln University, Lincoln University, PA, USA

**Keywords:** Forensic sciences, continuing education, educational requirements, online, web-based, certification

## Abstract

This study presents a detailed model and its subsequent assessment for the development and optimized delivery of continuing education for the forensic science community. Online continuing education is on the rise as this modality of content dissemination allows for accessibility, convenience, and affordability. The model, which includes an educational needs assessment, gap analysis, online content delivery and archival, was evaluated through the launch of a global training event/symposium series. The four-year study of over 6 000 participants representing >75 countries provides empirical support for the model based upon registration/attendance data and participant self-assessment surveys. Best practices are identified relative to content and format, operational excellence, technology, and financing of the event. Future directions along with opportunities to further enhance the model are also discussed.

**Key Points**
- A model is presented for the development, online delivery, and archival of compelling, cost effective continuing education content for the forensic science community.
- The model is evaluated based upon registration/attendance data and participant surveys.
- Best practices relative to content and format, operational issues, selection of technology and financing of the event are shared.

## Introduction

The proper application of modern forensic science is a central tenet in fostering justice, respect for human rights, democratic values, and the rule of law. It promotes social justice and equality through the introduction of unbiased, fact-based information to the criminal justice system. Critical to ensuring the quality and credibility of forensic science practice is undergraduate/graduate training, an established and accessible curriculum of continuing education and a programme of ongoing practitioner performance verification. Over the past decade, these three pillars of forensic science education have been the focus of various studies, programmes, and initiatives [1–10].

A key initiative in strengthening undergraduate and graduate education was the formation of a technical working group by the US National Institute of Justice (NIJ) for the purpose of recommending guidelines for forensic science educational programmes. Building on this initiative, the American Academy of Forensic Sciences (AAFS) with assistance from the NIJ subsequently established the Forensic Education Programs Accreditation Commission (FEPAC). Its charter was to develop standards and a formal evaluation/accreditation system for academic programmes. FEPAC accredited the first university programme in 2004. Since that time, 28 undergraduate forensic science programmes and 21 graduate forensic science programmes have been accredited by FEPAC (https://www.fepac-edu.org/accredited-universities).

Practitioner performance verification has similarly been under study with the aim of identifying opportunities for improvement. A particularly active area of investigation is the impact of cognitive bias on forensic science practice. Edmond et al. [11] recently reviewed the intersection of cognitive and forensic sciences providing insights that call into question common beliefs about human performance. They demonstrated through the work of White et al. [12] that education and experience without routine assessment fails to develop specialized expertise beyond the initial training. Without an ongoing programme of assessment and feedback, experienced practitioners were shown to perform no better than new analysts in the interpretation of evidence. While not the focus of any broad institution reform, there is a mounting body of literature that serves to educate, raise awareness, and provide a basis for revising conventional practices to enhance overall analyst performance.

Continuing education for forensic science practitioners is the primary focus of this work. Certification programmes such as the one administered by the American Board of Forensic Toxicology (ABFT) and the American Board of Criminalists (ABC) have been applauded and recognized for their rigour in establishing educational, training, and assessment requirements for forensic science professionals. Yet it can be a challenge to identify cost effective, accessible training programmes to meet these certification requirements. There is no programmatic approach to educational needs assessment and content deve­lopment. Nor is there an optimized delivery channel or central repository of available educational content. As a result, not all forensic toxicologists or forensic scientists are getting access to the training required to remain current in their field.

**Figure 1.**
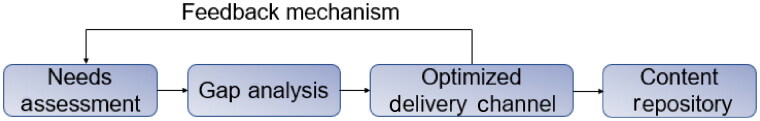
A proposed model for the development, delivery, and archival of continuing education content for forensic practitioners.

This study presents and evaluates a model for the development, online delivery, and archival of compelling, cost effective, continuing education content for forensic toxicologists with applicability to the broader forensic science community. The model consists of (1) a needs assessment for determining the value-added content required by the practitioner community, (2) a gap analysis to determine what needs are already being fulfilled through other ve­nues, (3) channel optimization in order to cost effectively deliver the content in an accessible, convenient format, (4) a feedback loop to allow for continuous improvement, and (5) a central repository to allow for re-purposing and enhancing content accessibility across global time zones (Figure 1).

## Methods

The model was evaluated through the launch and on-going expansion of a series of web-based/online forensic symposia named the *Forensic Online Symposium: Current Trends in Forensics & Forensic Toxicology* (www.forensicsymposium.org). The needs assessment was initially performed through a survey instrument which was later used in conjunction with input from Scientific Program Chairs who were well established in their respective disciplines, associated organizations, working groups, and/or standards consensus bodies. The Program Chairs also conducted the gap analysis and established the final scientific content for the training.

The content was delivered in an online, web-based format. Online continuing education is on the rise as this modality of disseminating information provides global accessibility, convenience, and affordability. An attendee registration system was used to provide quantitative data on attendance and attendee demographics. The initial event (May 2018) was held over 3 consecutive days, 3 h per day (9 am EST to 12 pm EST) and was focused on the discipline of forensic toxicology. Over the next 4 years, the Symposium was expanded to a series of week-long training events, each focused on a different discipline — forensic toxicology, forensic trace analysis, and seized drug analysis. All events were offered free of charge to participants with content pre-approved by the ABFT and the ABC for continuing education or recertification credit. Each day of the event was themed such that it represented a virtual Master Class — an in-depth examination of a specific topic presented by world-class practitioners having a unique mastery of the subject. Lecturers from diverse geographies were invited to provide a global perspective to the issue under study and a panel discussion was held at the conclusion of each day enabling interaction between attendees and the subject matter experts. Each successive event resulted in best practices and key learnings which were used in combination with attendee feedback to drive continuous improvement for subsequent iterations of the symposia. Following the live event, the slides and recorded presentations were made available free of charge for on-demand viewing at the convenience of the participants.

## Results

The *Forensic Online Symposium: Current Trends in Forensics & Forensic Toxicology* has delivered >100 h of training to nearly 6 000 forensic practitioners, researchers, academicians, and students in 75 countries. The initial event focused on forensic toxicology (May 2018), followed by forensic trace analysis (July 2020) and seized drug analysis (January 2021). Three years of attendance data for the forensic toxicology event are given in Table 1. In summary, 50% of registrants attended the live event while 20%−28% viewed the recorded content. This is consistent with expectations of a 40%−50% live attendance rate for web-based events as determined by recent benchmark studies [13]. The high percentage of on-demand views are largely due to registrants in time zones that are inconvenient for participation in the live event.

**Table 1.** Registration and attendance data for *Forensic Online Symposium: Current Trends in Forensics & Forensic Toxicology* 2018–2020.

Year	Registrants (*n*)	Live attendees (*n*, %)	Unique on-demand attendance (*n*, %)	Total attendance (*n*, %)
2018	1 000	530 (53%)	200 (20%)	730 (73%)
2019	1 200	650 (54%)	250 (21%)	900 (75%)
2020	1 400	710 (51%)	390 (28%)	1 100 (79%)

Geographically, 94% of registrants were from North America (64%), Europe (15%) and Asia (15%). Registrations from South America (4%), Australia (1%), and Africa (1%) combined represent the remaining 6%. The first forensic trace analysis event held in 2020 had 850 registrants and the 2021 seized drug exceeded 1 300 registrants. These observations suggest that the model can be expanded across multiple forensic science disciplines.

In a survey of event attendees, 90% of respondents indicated that their knowledge base improved through participation in the symposium and that the information presented was directly applicable to their professional role. Respondents cited the primary benefits of the event to include:

- Relevant, cost effective continuing education without the need to travel (increased accessibility).
- A global perspective of the topics discussed, and an increased understanding of how practitioners in other parts of the world were addressing similar concerns.
- The strengthening of a sense of community among global practitioners.

Through a combination of attendee feedback and the experience of implementing multiple iterations of the event, key learnings and best practices were identified relative to:

- Event content and format
- Operational excellence
- Technology as an enabler of distance learning, and
- Financial considerations

## Discussion

The inspiration for this educational model came from perceived shortcomings of face-to-face confe­rences combined with the challenge of meeting professional continuing education requirements. It was observed that face-to-face conferences were often attended by the same individuals every year. Those unable to participate were frequently limited by the cost of attendance (registration, workshops, short courses, travel, etc.) or by time constraints arising from professional or personal responsibilities. Furthermore, the time allocated for oral presentations was typically limited to 10 − 15 min. Although this was adequate for providing a summary of the work undertaken, it was seldom sufficient to explore the topic in any detail. Short courses and workshops did offer more in-depth treatment but at an additional cost. The objective of this study was to develop, implement and evaluate a model designed to provide cost effective, accessible, in-depth continuing educational content.

### Formulating the model: current and future state

**Figure 2.**
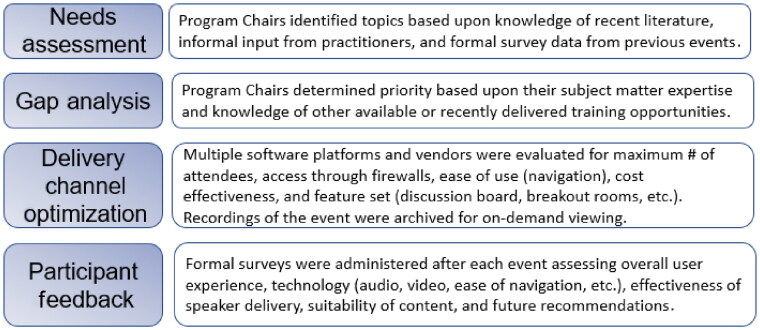
Application of the model to the *2020 Forensic Online Symposium*: *Current Trends in Forensics & Forensic Toxicology.*

The process began with a needs assessment and gap analysis to determine what constituted compelling content, i.e. topics that were timely, relevant, and had not been previously addressed in sufficient depth or widely communicated. This was done using a survey instrument in combination with the specialized knowledge of the Program Chairs (Figure 2). The rudimentary approach taken in this study could be improved upon by a more formal, rigorous, process driven, needs assessment and gap analysis institutionalized by one of the analyst certifying bodies, professional forensic organizations, or other oversight committees. If conducted on some regular basis, one could envision codification of a standardized curriculum of continuing education for forensic scientists based upon discipline and experience level. This would provide direction to the many non-profit and commercial organizations offering courses and webinars to ensure the delivery of value-added content. It could also serve as a performance measure for employee development as part of organizations’ balanced scorecard [14].

The goal of delivery channel optimization is accessibility. Ideally, participants would have the opportunity to access the same value-added content in a face-to-face venue, live online, or recorded on-demand. This study focused largely on the latter two options as face-to-face delivery has been the existing paradigm. As such, there were considerable learnings and best practices relative to technology as an enabler that will be discussed later in this article. One aspect of delivery that remains a challenge is the wide diversity of time zones. It is impossible to find a time period that is convenient in all time zones around the world. Since its inception, the *Forensic Online Symposium* has always been held from 9 am to 12 pm EST so there is merit in exploring if this time window is optimal as measured by attendance. Another aspect of accessibility that has not been addressed is language diversity. An earlier iteration of the symposium explored the use of closed captioning in attempts to address this issue, however captioning of scientific terms was largely unsuccessful making the text unintelligible. A recent meeting of the International Alliance of Clinical and Forensic Toxicologists (IACFT, https://www.iacft.online/) is believed to be the first online event in forensic toxicology that was broadcast in multiple languages and may offer best practices for future global meetings.

Live, on-demand accessibility could be facilitated through the development of a central content reposi­tory. At the current time, all presentations, posters, and recordings from the 2020 symposium are available on-demand with plans to expand the archive to other previous and future events in the series. Ideally, there would exist a comprehensive national central catalogue of continuing education courses inclusive of all delivery mechanisms. Once presented, new content would be maintained in a central archived repository ensuring ready access while reducing the development of redundant training material by various disparate sources. This would be greatly facilitated if, as suggested previously, a standard curriculum based upon a rigorous, regularly updated needs assessment and gap analysis were to be undertaken. A feedback mechanism for continuous improvement could easily be inserted at any point in the model. If implemented as envisioned, the result would be a programme of priori­tized continuing education content pre-approved by analyst certifying bodies. There would be a directory of courses delivered through multiple channels, supported through a central repository of archived material to maximize accessibility.

### Best practices: event content and format

The final format of the online event was the result of multiple iterations and direct feedback from attendees. Each event spanned 5 days, 3 h per day allowing attendees the opportunity to learn from their desktop without experiencing excessive computer fatigue or losing an entire day’s productivity. Each day was themed with speakers selected from across the globe to provide geographic perspective on the topic of discussion. Presenters were asked to coordinate their lectures (40 − 60 min in duration) to ensure a progressive flow of material without gaps or unnecessary redundancy. Presentation material was made available for download at the start of each lecture so that participants could make notes or follow along with the speaker. A recording of the event was released immediately following the last presentation of the day. This enabled those who were unable to attend the live event to view the lectures on the same day that they were initially presented.

Presenters were requested to turn on their came­ras when speaking to establish a more personal connection with the audience. A short question-and-answer (Q&A) period followed each lecture. Additionally, a 30-min panel discussion that included all of the presenters was held at the conclusion of each day to address unanswered questions or expostulate on the broader theme of the day. Attendees were encouraged to continue the dialogue beyond the conclusion of the day’s live programme *via* online discussion boards. These efforts to encourage audience interaction were specifically designed to establish a virtual sense of community among attendees.

### Best practices: operational excellence

Operational excellence strives for a superior, trouble free attendee experience. It requires strong teamwork among all stakeholders, clear roles and responsibili­ties, and an equally high level of commitment among all involved to the success of the programme. It also requires extensive contingency planning so that should the unexpected occur, there is no disruption to the end user experience.

It is recommended, for example, to have one-on-one familiarization sessions with each speaker to confirm that the computer, operating system, and accessories (camera, microphone, speakers, etc.) that will be used for the event are compatible with the hosting platform. It is suggested that these sessions also be conducted using the same network and network connection (wireless *vs.* local area network) that will be used on the day of the event to ensure sufficient speed, bandwidth, and reliability.

Emergency contact information should be collated for all speakers and conversely speakers should be provided with an alternate call-in number if the event relies on voice over internet protocol (VOIP) in case of unanticipated network disruptions. It is recommended that speakers submit presentations to the event host in advance so should something prevent the speaker from sharing slides during the event, the host can broadcast the material to the audience. Another best practice is to have all spea­kers record presentations in advance so should something interfere with the speaker or the speaker’s network, the recordings can be broadcast to the audience *via* the event host. It is also suggested that each presenter provide contact information for (and prepare) a contingency speaker in case a business-critical issue such as a court testimony arises necessitating a last-minute cancellation.

Technology support is equally important for attendees to ensure a positive experience. A best practice is to provide an automated system compati­bility test for all attendees prior to the event as well as a recorded demonstration of how to navigate the online venue and its key features. A frequently asked questions (FAQ) document readily accessible on the site and information relative to different computer types and operating systems are of great importance. Not everyone will have a Windows®-based computer and different systems can have their own unique challenges. Finally, ensure that real time technical support is available throughout the event both for speakers as well as the attendees. Providing an online experience with clean visuals, clear audio, and uninterrupted content streaming are key to a positive and enjoyable learning experience.

### Best practices: technology

Technology is a core enabler for creating an optimized delivery channel for continuing education. When evaluating the various software platforms and hosting service options available, it is critical to remember that form follows function. It is critical to determine the core capabilities that are required for the event, i.e. registration tracking, certificate generation, live technical support, archival capabili­ties, maximum number of attendees, etc. It is also important to define the target audience. It is well known that certain platforms cannot penetrate the firewalls of all organizations or agencies. Likewise, not all platforms are accessible in all countries. Once the core requirements have been defined, a platform could be selected to offer those capabilities. Beware of feature creep — the more feature rich the platform, the more it will cost. Having a well-defined requirements document prepared in advance will facilitate balancing cost and capabilities. Finally, always prioritize ease of use. It is of paramount importance that attendees can seamlessly register for the event and navigate the online environment.

### Best practices: financials

Although web-based events are less expensive to support than face-to-face meetings, there are still costs involved: platform licenses, hosting fees, server space, site customization, video rendering, and technical support, just to name a few. Some platforms also charge a fee per attendee or are based on the duration of the event. In general, feature set determines costs, so it is important to distinguish between a requirement and a desired capability. Other hidden costs to consider include the development of promotional material such as schedules, abstract books, proceedings, and other marketing costs to let potential attendees know about the existence of the event.

The costs of hosting an event can be funded in several ways including membership dues in the case of organizational events, donations, registration fees, sponsorships, or a combination of all of the above. The important thing to remember, regardless of where the funding is coming from, is that the funding source is looking for value in return. Sponsors require a return on marketing investment, attendees expect to receive tangible value for their registration fee, even donors like to know how their money is being spent and the value it is providing to the community. Value creation for attendees, sponsors, donors, and any other stakeholders is a critical concept to understand and master for continued success.

### Conclusions and future directions

This study presents empirical support and best practices for an enhanced model for the development, delivery, and archival of continuing education content for forensic professionals. The model initially implemented for forensic toxicology has been extended to a week-long series of events addressing topics related to forensic trace analysis and the chara­cterization of seized drugs. Future implementations of this model will strive for more rigorous and process driven needs assessment and gap analysis engaging a larger cross-section of stakeholders.

The current paradigm espouses preference for face-to-face content delivery largely due to enhanced social and networking opportunities. The inherent value of this modality is recognized but as discussed previously, it is not without its shortcomings particularly relative to accessibility. The results obtained in this study demonstrate that web-based mechanisms offer more cost effective, accessible content delivery than is possible in a face-to-face format alone. Future work will focus on continuing to improve online accessibility both geographically (across time zones) and culturally (language). More advanced technologies will be explored to enable features such as multiple streaming sessions, real time translation, and real time audio Q&A all with the aim of increasing the human element and fostering a stronger sense of engagement among participants. It is suggested that the optimized content delivery channel for professional continuing education would include some hybrid of all three approaches: live face-to-face events, live online events and archived on-demand viewing (which also requires future work in the establishment of a single resource/repository for archived material). Self-assessment by participants found the online model effective in fulfilling individual learning objectives while meeting the standards of analyst certifying bodies such as the ABFT and the ABC.
